# Transitions from cigarette smoking by geolocation among US adults: Results from waves 1-6 of the Population Assessment of Tobacco and Health (PATH) study 2013–2021

**DOI:** 10.18332/tid/226559

**Published:** 2026-10-28

**Authors:** Jenny E. Ozga, Melissa H. Abadi, Nancy C. Jao, John H. Kingsbury, Michael J. Parks, Alayna P. Tackett, Wubin Xie, Shu Xu, Jing Zhang, Cassandra A. Stanton

**Affiliations:** 1 Public HealthWestatBethesdaUnited States; 2 Pacific Institute for Research and EvaluationLouisvilleUnited States; 3 Department of PsychologyRosalind Franklin University of Medicine and ScienceNorth ChicagoUnited States; 4 National Institute on Drug Abuse, National Institutes of HealthBethesdaUnited States; 5 Kelly Government SolutionsBethesdaUnited States; 6 Center for Tobacco Research, The Ohio State University Wexner Medical Center, The James Comprehensive Cancer CenterColumbusUnited States; 7 Lee Kong Chian School of Medicine, Nanyang Technological UniversitySingaporeSingapore; 8 School of Global Public HealthNew York UniversityNew YorkUnited States; 9 College of NursingUniversity of KentuckyLexingtonUnited States

**Keywords:** rural, urban, polytobacco, cessation, e-cigarette

## Abstract

**Introduction:**

Adults in rural US areas experience higher rates of tobacco use and related disease. They also have lower cigarette cessation success than those in non-rural areas, but it is unknown how transitions from exclusive cigarette use to other product use differ by geolocation. This study examined longitudinal associations between geolocation (urban, suburban, town, rural) and transitions from exclusive cigarette smoking.

**Methods:**

We conducted a secondary analysis of pooled wave-pair observations from US adults participating in Waves (W) 1–6 of the Population Assessment for Tobacco and Health Study (2013–2021) who had exclusive past 30-day cigarette smoking at the wave-pair baseline and one of the following at the wave-pair follow-up: 1) continued exclusive cigarette smoking, 2) continued smoking with other added tobacco products, 3) switched to exclusive e-cigarette use, or 4) quit tobacco completely (reference group). Survey-weighted random-intercept multinomial logistic regressions were conducted, adjusting for sociodemographics, smoking-related factors, and survey wave.

**Results:**

Of 13634 wave-pairs (32.3% suburban, 28.3% urban, 27.3% rural, 12.1% town), 90.8% continued smoking (73.7% exclusively smoking cigarettes, 17.1% with other added tobacco products), 1.2% switched to e-cigarettes, and 8.0% quit tobacco completely. Town (adjusted relative risk ratio, ARRR=1.52; 95% CI: 1.10–2.10) or rural (ARRR=1.34; 95% CI: 1.05–1.69) adults were significantly more likely to continue exclusive smoking than urban adults, with no significant associations between geolocation and other tobacco transitions.

**Conclusions:**

Living in town or rural (vs urban) US areas was associated with an increased likelihood of continued exclusive cigarette smoking compared to quitting tobacco, suggesting that targeted interventions and policies are needed to support these populations.

## Introduction

Compared to people living in non-rural areas of the United States (US), people living in rural areas experience disproportionately higher rates of cigarette and smokeless tobacco use^1^ and related diseases, including chronic obstructive pulmonary disease^2^ and lung cancer^3^. Findings are mixed for e-cigarette use, with some studies showing lower rates of e-cigarette use in rural areas and others not showing differences by geolocation^1,4,5^. People living in rural areas also make fewer cigarette quit attempts^6^ and are less likely to have cessation success compared to those in non-rural areas^7,8^, but it is unknown how transitions from exclusive cigarette smoking to smoking with other added nicotine or tobacco products or exclusive use of e-cigarettes may differ by geolocation. This is important because adults may transition from cigarettes to e-cigarettes for a variety of reasons, including smoking cessation^9,10^, but differences across geolocation are not well-studied.

Most published work regarding geolocation and tobacco use has dichotomized areas in rural (or non-urban) and non-rural (or urban) categories. However, there are various ways to classify geolocations that take into account different geographical characteristics^11-13^. In a recently published study using nationally representative data of adults in the Population Assessment of Tobacco and Health (PATH) Study, two geolocation classifications and their associations with cigarette, e-cigarette, and smokeless tobacco use were directly compared^14^; one geolocation classification was dichotomous (urban, non-urban), and the other was divided into four categories (urban, suburban, town, rural). Although the two classifications had overlap and were similarly associated with tobacco use, the 4-category classification was found to be a more sensitive measure that revealed misclassifications and detected more differences, particularly for respondents classified as being in town areas. Specifically, although the dichotomous definition classified ~90% of ‘town’ respondents as urban, study findings suggested that ‘town’ respondents may be more similar to ‘rural’ respondents in terms of their smokeless tobacco use.

The present study examined associations between four unique US geolocations (urban, suburban, town, or rural) and transitions from exclusive cigarette smoking among a nationally representative sample of US adults in the PATH Study cohort. Data from Waves 1–6 (2013–2021) of the PATH Study using a series of adjacent wave-pairs (e.g. Wave 1 to Wave 2) were used to examine transitions over a period of 1–2 years from exclusive cigarette smoking to: 1) continued exclusive smoking; 2) continued smoking with other added tobacco products; 3) exclusive e-cigarette use; and 4) quitting tobacco completely. Consistent with the PATH Study and the US Food and Drug Administration’s regulatory framework, the term ‘tobacco products’ for our study includes cigarettes, e-cigarettes, cigars, smokeless tobacco, hookah, snus, and pipe tobacco.

## Methods

### Study design and sample

We conducted an analysis using restricted-use data from adults (aged ≥18 years) in Waves (W) 1–6 (2013–2021) of the PATH Study^15^, a national longitudinal cohort survey of US youth and adults. W1-W4 were conducted annually, whereas the intervals between W4/W5 and W5/W6 were approximately two years. Details about the PATH Study design, methods, and instruments are publicly available^15^. Data for W1-W5 were collected in-person. Due to COVID-19, W6 data were collected with a combination of telephone and in-person interviews. There were 32320 adults included in the W1 cohort (2013–2014). We created an analytic dataset that included up to five wave-pair observations for each respondent: (baseline follow-up) W1-W2, W2-W3, W3-W4, W4-W5, and W5-W6. Analyses were restricted to consecutive wave-pairs because non-consecutive waves could encompass multiple unobserved tobacco use transitions, resulting in ambiguity regarding the timing and sequence of behavior change. The current analysis was limited to adults who exclusively smoked cigarettes in the past 30 days during a baseline wave, were categorized as having one of the four cigarette use transitions of interest, and were not missing data on any study measures (n=4774 respondents; n=13634 observations; Supplementary file Figure 1). The primary analysis was restricted further to adults who had completed all six survey waves in order to obtain nationally representative estimates using W6 all-waves survey weights. This study qualified as exempt from ethics review by the Westat Institutional Review Board and was conducted following STROBE guidelines.

### Measures

#### Geolocation

Geolocation categorizations were provided by the PATH Study^15^ at W1-W5 (wave-pair baseline) and came from the National Center for Education Statistics (NCES). Respondents were categorized as living in urban, suburban, town, or rural areas of the US categories were assigned to individual respondents at the US census block level using 2021 census block definitions by the PATH Study. Respondents’ geolocations were coded to match 12-level locale classifications from the NCES^16^ prior to being collapsed by the PATH Study into the four categories listed^15^.

#### Past 30-day tobacco use

Respondents were included in a wave-pair observation if they exclusively smoked cigarettes in the past 30 days (P30D) at the baseline wave for that wave-pair and either: 1) continued exclusive cigarette smoking; 2) continued smoking with other added tobacco products; 3) switched to exclusive e-cigarette use; and 4) quit tobacco entirely at the follow-up wave for that wave-pair. Exclusive cigarette smoking was defined as cigarette smoking in the P30D and no use of e-cigarettes, smokeless tobacco, snus, hookah, cigars, or pipe. Our primary outcome was a 4-category measure that indicated whether respondents in P30D: 1) continued exclusive cigarette smoking (cigarette smoking and no other tobacco product use); 2) added use of another tobacco product (use of cigarettes and at least one other tobacco product); 3) switched completely to e-cigarettes (e-cigarette use and no other tobacco product use, including no use of cigarettes); or 4) quit tobacco completely (no tobacco product use, including no use of cigarettes).

#### Covariates

Sociodemographic covariates from each wave-pair baseline included age, sex, race, ethnicity, education level, total household income, and US region of residence. Missing data on age, sex, and race were imputed as described in the PATH Study Restricted Use Files User Guide^15^.

Other covariates that were included in the analysis were survey wave, absolute cigarette risk perceptions (dichotomized as not at all/slightly/somewhat or very/extremely harmful), noticing warnings on cigarette packs in the P30D (yes or no), cigarette smoking frequency (daily or nondaily), cigarette dependence (minutes from waking to first cigarette), and cigarette pack-years from baseline waves. Cigarette pack-years were calculated using respondents’ self-reported age of cigarette onset, current age, and cigarettes smoked per day^17^. Cigarette pack-years and minutes of waking to first cigarette were minorized by reassigning values from the 99th percentile to the top and bottom 1% of values to reduce undue influence of outliers^18^. Minutes to first cigarette was used as the measure of cigarette dependence because cigarette pack-years, which includes cigarettes smoked per day, were modeled separately.

### Statistical analysis

Weighted descriptive statistics and random-intercept multinomial logistic regression were used to evaluate associations between geolocation and P30D cigarette use transitions across wave-pairs. The multinomial logistic regression model estimated the effects of geolocation on the relative risk of P30D: continuing exclusive cigarette smoking, continuing smoking with other added tobacco products, or switching to exclusive e-cigarette use, compared to quitting tobacco completely (no tobacco use, reference). We estimated an unadjusted model and a covariate-adjusted model, reporting relative risk ratios (RRRs) and adjusted RRRs (ARRRs) with 95% confidence intervals (CIs), respectively. All covariates had variance inflation factors (VIFs) <5, indicating that multicollinearity was not a concern. Adjusted predicted probabilities were also derived to show the likelihood of each tobacco use transition by geolocation, controlling for covariates.

Analyses were weighted using the W6 all-waves survey weights, which included full sample and 100 replicate weights, to produce nationally representative estimates that account for the complex survey design and attrition across waves. Variances were computed using the balanced repeated replication (BRR) method with Fay’s adjustment set to 0.3 to obtain valid standard errors. Details on how replicate weights were constructed by the PATH Study can be found in the PATH Study Restricted Files User Guide^15^. All analyses were conducted using Stata/MP 17.0.

#### Missing data analysis

Wave 6 all-waves weights addressed missingness related to respondents who were lost to follow-up across waves in our primary analysis, which included only complete cases. However, survey weights did not address exclusion of 15.6% of observations based on missing data on one or more survey measures. We therefore examined sample characteristics among adults who exclusively smoked cigarettes in the P30D at baseline based on whether respondents were included (84.4%) or excluded (15.6%) from the analytic sample due to missingness on study measures. We conducted this analysis to determine whether differences between the analytic (complete case) and excluded sub-samples may limit generalizability of findings.

#### Sensitivity analysis

Given the large number of respondents who were removed from weighted analyses due to missing W6 all-waves survey weights (i.e. they did not complete all 6 waves of data collection), we conducted an unweighted (not nationally representative) sensitivity analysis that included adult respondents who were lost to follow-up but contributed one or more wave-pair observations. Adding these data increased the total number of unique respondents (n=8459) and wave-pair observations (n=20628).

## Results

### Sample characteristics

Shown in Table 1, across all wave-pair observations (n=13634), respondents were split evenly on sex but were mostly White (78.9%) and non-Hispanic (88.7%). Most respondents resided in suburban areas (32.3%), followed by urban (28.3%), rural (27.3%), and town (12.1%). There were more respondents from the South (40.2%) region, followed by the Midwest (23.6%), Northeast (18.4%), and West (17.8%). There was a relatively even distribution of age among respondents aged ≥25 years; however, only 6.1% of the sample comprised young adults (age 18–24 years). Most respondents had completed some college or lower education (87.4%), with only 12.6% reporting a Bachelor’s degree or higher. Most respondents (67.1%) reported annual household income of ≤$50000. Approximately three-quarters of the sample reported that they believe cigarettes are very/extremely harmful to health (72.6%) and noticed warnings on cigarette packs in the past 30 days (77.0%). The average cigarette pack-years were 21.2 (SD=23.4) and minutes to first cigarette after waking were 95.3 (SD=176.0). Most respondents continued exclusive cigarette smoking (73.7%), followed by continued smoking with other added tobacco products (17.1%), quitting tobacco completely (8.0%), and switching to exclusive e-cigarette use (1.2%).

**Table 1:** Baseline wave sociodemographic and cigarette use characteristics among adults who exclusively smoked cigarettes in the past 30 days at baseline, Waves 1–6 of the Population Assessment of Tobacco and Health (PATH) Study, 2013–2021 (N=4774)

Characteristics	Total ^b^ (N=13634)	Transition type from baseline to follow-up ^a^			
		Continued exclusive smoking (N=10080)	Continued smoking with other added tobacco products (N=2392)	Switched to e-cigarettes (N=166)	Quit tobacco completely (N=996)
	Weighted row % or (Weighted mean) (95% CI)				
**Residence**					
Urban	28.3 (24.7–32.2)	71.4 (69.3–73.3)	18.2 (16.6–20.0)	1.1 (0.8–1.5)	9.3 (8.1–10.7)
Suburban	32.3 (27.9–37.0)	72.3 (70.3–74.1)	16.7 (15.3–18.3)	1.4 (1.0–2.0)	9.6 (8.2–11.3)
Town	12.1 (9.2–15.7)	76.0 (73.3–78.5)	17.4 (15.1–20.0)	1.3 (0.9–2.0)	5.3 (4.1–6.8)
Rural	27.3 (23.1–32.0)	76.9 (75.4–78.3)	16.3 (14.8–17.9)	1.0 (0.7–1.5)	5.8 (5.0–6.7)
**US region**					
Northeast	18.4 (16.5–20.4)	74.6 (72.0–77.0)	16.9 (14.4–19.7)	1.0 (0.6–1.6)	7.5 (5.8–9.8)
Midwest	23.6 (22.0–25.4)	75.4 (73.7–77.1)	16.7 (15.3–18.3)	1.1 (0.8–1.6)	6.7 (5.8–7.8)
South	40.2 (37.6–42.9)	73.0 (71.5–74.5)	17.4 (16.1–18.7)	1.3 (0.9–1.7)	8.3 (7.2–9.7)
West	17.8 (15.9–19.8)	72.2 (69.8–74.4)	17.3 (15.4–19.3)	1.4 (1.0–1.8)	9.2 (7.7–10.9)
**Age** (years)					
18–24	6.1 (5.5–6.7)	54.1 (50.5–57.6)	33.8 (30.6–37.2)	2.2 (1.4–3.5)	9.9 (7.9–12.3)
25–39	32.6 (31.0–34.2)	65.4 (63.5–67.2)	23.2 (21.4–25.0)	1.8 (1.4–2.3)	9.7 (8.6–10.9)
40–54	31.2 (29.5–33.1)	72.0 (76.5–79.5)	15.4 (13.9–17.1)	1.0 (0.7–1.4)	5.6 (4.7–6.6)
≥55	30.1 (28.3–31.9)	82.3 (80.7–83.8)	8.9 (7.9–10.0)	0.6 (0.4–1.0)	8.2 (6.8–9.8)
**Sex**					
Male	49.1 (47.5–50.6)	72.1 (70.6–73.4)	19.2 (17.9–20.5)	1.2 (0.9–1.5)	7.6 (6.7–8.6)
Female	51.0 (49.4–52.5)	75.3 (74.0–76.6)	15.1 (14.1–16.2)	1.2 (1.0–1.5)	8.3 (7.4–9.3)
**Race**					
White	78.9 (77.4–80.3)	73.9 (72.9–74.9)	16.7 (15.7–17.7)	1.3 (1.1–1.6)	8.1 (7.4–9.0)
Black	14.7 (13.6–16.0)	74.8 (72.2–77.2)	18.7 (16.8–20.6)	0.7 (0.4–1.4)	5.8 (4.7–7.2)
Other/multiple	6.3 (5.6–7.2)	68.8 (65.0–72.3)	19.2 (16.5–22.2)	1.3 (0.7–2.7)	10.7 (8.3–13.8)
**Ethnicity**					
Hispanic	11.3 (10.3–12.5)	69.7 (66.0–73.1)	16.8 (13.9–20.1)	0.6 (0.4–1.1)	12.9 (10.8–15.4)
Non-Hispanic	88.7 (87.6–89.7)	74.3 (73.3–75.2)	17.2 (16.3–18.0)	1.3 (1.1–1.5)	7.3 (6.7–8.1)
**Education level**					
< High school	26.5 (25.1–28.0)	75.8 (74.2–77.3)	17.0 (15.7–18.4)	0.8 (0.5–1.2)	6.5 (5.3–7.8)
High school degree/GED	27.8 (26.3–29.4)	74.5 (74.6–78.2)	16.1 (14.7–17.7)	1.1 (0.8–1.4)	6.4 (5.2–7.8)
Some college/ Associate’s degree	33.1 (31.5–34.7)	71.7 (70.1–73.2)	18.6 (17.3–20.0)	1.5 (1.1–2.0)	8.3 (7.4–9.3)
Bachelor’s or higher	12.6 (11.4–13.8)	68.8 (65.9–71.5)	15.6 (13.6–17.9)	1.7 (1.1–2.7)	13.9 (11.6–16.6)
**Household income** ($)					
≥100000	7.3 (6.3–8.5)	69.4 (65.4–73.1)	16.0 (12.9–19.8)	2.1 (1.3–3.6)	12.5 (9.9–15.6)
50000–99999	20.0 (18.7–21.3)	73.7 (71.3–75.8)	15.3 (13.4–17.5)	1.6 (1.2–2.3)	9.4 (7.8–11.2)
25000–49999	24.5 (23.4–25.6)	74.1 (72.1–76.0)	17.2 (15.6–18.9)	1.3 (0.9–1.9)	7.4 (6.3–8.7)
<25000	42.6 (40.8–44.3)	74.3 (72.9–75.6)	18.3 (17.2–19.4)	0.9 (0.7–1.2)	6.6 (5.7–7.6)
**Smoking frequency**					
Daily	80.2 (78.9–81.5)	76.9 (75.9–77.7)	17.4 (16.5–18.3)	1.1 (0.9–1.3)	4.7 (4.2–5.2)
Non-daily	19.8 (18.5–21.1)	61.1 (58.8–63.3)	15.9 (14.2–17.8)	1.8 (1.3–2.4)	21.3 (19.1–23.6)
**Mean cigarette pack-years**	(21.2) (20.3–22.0)	(21.9) (21.0–22.8)	(18.3) (17.3–19.3)	(16.8) (13.0–20.6)	(20.9) (18.6–23.3)
**Mean minutes to first cigarette upon waking**	(95.3) (89.2–101.5)	(84.2) (77.8–90.7)	(80.0) (72.6–87.4)	(129.8) (101.5–158.1)	(226.1) (204.5–247.7)
**Cigarette risk perception**					
Not at all/slightly/ somewhat harmful	27.5 (26.3–28.6)	75.8 (74.1–77.4)	16.9 (15.6–18.4)	0.7 (0.5–1.0)	6.6 (5.6–7.8)
Very/extremely harmful	72.6 (71.4–73.7)	73.0 (71.9–74.0)	17.2 (16.2–18.2)	1.4 (1.2–1.7)	8.5 (7.7–9.4)
**Noticed warnings on cigarette packs in the past 30 days**	77.0 (75.7–78.2)	74.1 (73.1–75.1)	17.2 (16.3–18.2)	1.2 (1.0–1.5)	7.4 (6.7–8.2)

a ‘Continued exclusive smoking’: adults who continued exclusively smoking cigarettes at follow-up. ‘Continued smoking with other added tobacco products’: adults who continued smoking cigarettes and reported past 30-day use of at least one other nicotine/tobacco product at follow-up. ‘Switched to e-cigarettes’: adults who reported exclusive e-cigarette use at follow-up. ‘Quit tobacco completely’: adults who reported no past 30-day use of any nicotine/tobacco product (including cigarettes) at follow-up.

b Some respondents contributed multiple wave-pair observations.

### Analytic sample versus excluded sample due to missing study measures

When we compared sample characteristics among adults who exclusively smoked cigarettes in the P30D at baseline based on whether respondents were included (84.4%) or excluded (15.6%) from the analytic sample due to missingness on study measures, we found that the excluded sample less often continued exclusive smoking or continued smoking with other added tobacco products, and more often quit tobacco completely across wave-pairs than the analytic sample (Supplementary file Table 1). Respondents excluded from the analytic sample because of missing study measures, were also more often from urban or suburban areas relative to town or rural, were more often located in the West, and less often in the Midwest regions of the US. Excluded respondents were also younger, more educated, had higher household income, were more frequently Hispanic, and less frequently White. For cigarette-related characteristics, excluded respondents had: significantly higher pack-years of cigarette smoking; waited significantly longer upon waking to smoke their first cigarette; reported non-daily cigarette smoking and viewing cigarettes as very/extremely harmful to health more often; and noticed warnings on cigarette packs in the P30D less often compared to the analytic sample.

### Unadjusted associations between geolocation and cigarette use transitions

Shown in Table 1, town had the lowest proportion of respondents who quit tobacco completely (5.3%), followed by rural (5.8%), urban (9.3%), and suburban (9.6%). Rural areas had the largest proportion of respondents who continued exclusive cigarette smoking (76.9%), followed by town (76.0%), suburban (72.3%), and urban (71.4%). Transitions to continued smoking with other added tobacco products or exclusive e-cigarette use were similar across geolocations.

As shown in Table 2, there was a significant unadjusted association between geolocation and cigarette use transitions. Rural (vs urban) was associated with increased risk of continued exclusive cigarette smoking (RRR=1.88; 95% CI: 1.46–2.41; p<0.001) and continued smoking with other added tobacco products (RRR=1.45; 95% CI: 1.07–1.97; p=0.018) compared to quitting tobacco. Town (vs urban) was also associated with increased risk of continued exclusive cigarette smoking (RRR=2.09; 95% CI: 1.48–2.94; p<0.001), continued smoking with other added tobacco products (RRR=1.74; 95% CI: 1.19–2.53; p=0.005), or switching to exclusive e-cigarette use (RRR=2.17; 95% CI: 1.18–4.00; p=0.014) compared to quitting tobacco.

**Table 2:** Unadjusted and adjusted weighted multinomial logistic regressions examining associations between geolocation and cigarette use transitions (reference quitting all tobacco) among adults who exclusively smoked cigarettes in the past 30 days at baseline, Waves 1–6 of the Population Assessment of Tobacco and Health (PATH) Study, 2013–2021 (N=4774)

Variables	Transition type from baseline to follow-up					
	Continued exclusive smoking		Continued smoking with other added tobacco products		Switch to e-cigarette use	
	RRR	ARRR (95% CI)	RRR	ARRR (95% CI)	RRR	ARRR (95% CI)
**Residence**						
Urban (ref.)						
Suburban	0.98	0.99 (0.79–1.26)	0.87	0.94 (0.73–1.22)	1.29	1.27 (0.67–2.37)
Town	**2.09**	1.52 (1.10–2.10)	**1.74**	1.27 (0.88–1.83)	**2.17**	1.61 (0.88–2.94)
Rural	**1.88**	1.34 (1.05–1.69)	**1.45**	1.16 (0.86–1.56)	1.38	1.08 (0.59–1.99)
**US region**						
Northeast (ref.)						
Midwest	1.16	0.96 (0.70–1.32)	1.12	0.87 (0.58–1.31)	1.28	1.11 (0.52–2.36)
South	0.89	0.75 (0.55–1.03)	0.95	0.76 (0.51–1.13)	1.14	1.19 (0.58–2.41)
West	0.77	0.95 (0.70–1.31)	0.85	0.98 (0.64–1.51)	1.14	1.38 (0.68–2.79)
**Age** (years)						
18–24 (ref.)						
25–39	**1.42**	1.14 (0.83–1.56)	**0.80**	0.64 (0.46–0.88)	0.72	0.56 (0.27–1.15)
40–54	**3.28**	2.13 (1.51–3.00)	**0.90**	0.59 (0.41–0.86)	0.66	0.48 (0.22–1.03)
≥55	**2.54**	1.59 (1.08–2.35)	**0.36**	0.22 (0.14–0.34)	**0.25**	0.19 (0.08–0.47)
**Sex**						
Male (ref.)						
Female	1.00	1.01 (0.84–1.22)	**0.69**	0.71 (0.57–0.88)	0.98	0.94 (0.62–1.41)
**Race**						
White (ref.)						
Black	**1.43**	1.47 (1.09–1.98)	**1.65**	1.72 (1.29–2.30)	0.73	0.85 (0.35–2.07)
Other/multiple	**0.66**	0.87 (0.62–1.21)	0.90	0.93 (0.66–1.32)	0.79	0.86 (0.35–2.11)
**Ethnicity**						
Hispanic	**0.50**	0.92 (0.69–1.24)	**0.56**	0.85 (0.62–1.16)	**0.25**	0.30 (0.12–0.74)
Not Hispanic (ref.)						
**Education level**						
<High school (ref.)						
High school/ degree/GED	1.04	1.06 (0.81–1.39)	0.93	0.95 (0.72–1.24)	1.47	1.28 (0.68–2.41)
Some college/ Associate’s degree	**0.71**	0.84 (0.65–1.08)	0.82	0.96 (0.72–1.28)	1.70	1.34 (0.70–2.59)
Bachelor’s or higher	**0.37**	0.70 (0.50–0.97)	**0.40**	0.79 (0.54–1.18)	1.11	0.96 (0.40–2.30)
**Household income** ($)						
≥100000	**0.47**	0.87 (0.60–1.26)	**0.45**	0.78 (0.48–1.26)	1.44	1.69 (0.67–4.27)
50000–99999	**0.69**	0.95 (0.72–1.24)	**0.60**	0.79 (0.59–1.05)	1.39	1.41 (0.80–2.48)
25000–49999	0.87	0.93 (0.72–1.19)	0.83	0.85 (0.64–1.12)	1.31	1.21 (0.74–1.98)
<25000 (ref.)						
**Smoking frequency**						
Daily	**6.33**	4.01 (3.18–5.04)	**5.28**	3.57 (2.73–4.66)	**2.67**	2.05 (1.08–3.90)
Non-daily (ref.)						
**Mean cigarette pack-years**	1.00	1.00 (1.00–1.00)	**0.99**	1.00 (0.99–1.00)	0.99	1.00 (0.99–1.01)
**Mean minutes to first cigarette upon waking**	**1.00**	1.00 (1.00–1.00)	**1.00**	1.00 (1.00–1.00)	**1.00**	1.00 (1.00–1.00)
**Cigarette risk perception**						
Not at all/slightly/ somewhat harmful (ref.)						
Very/extremely harmful	**0.75**	0.89 (0.73–1.08)	**0.76**	0.85 (0.68–1.05)	**1.75**	1.74 (0.95–3.18)
**Noticed warnings on cigarette packs in the past 30 days**	**1.41**	1.34 (1.09–1.64)	**1.40**	1.30 (1.06–1.59)	1.42	1.29 (0.73–2.28)

ARRR: adjusted relative risk ratio. CI: confidence interval. Bolded values denote statistical significance at p<0.05.

### Adjusted associations between geolocation and cigarette use transitions

As shown in Table 2, there was a significant association between geolocation and cigarette use transitions after adjusting for covariates. Compared to urban, town (ARRR=1.52; 95% CI: 1.10–2.10; p=0.013) and rural (ARRR=1.34; 95% CI: 1.05–1.69; p=0.017) respondents had significantly increased risk for continued exclusive cigarette smoking relative to quitting tobacco. There were no significant adjusted associations between geolocation and transitions to continued smoking with other added tobacco products or exclusive e-cigarette use.

Predicted probabilities adjusted for covariates (Figure 1; and Supplementary file Table 2) indicated that continued exclusive cigarette smoking was highest in town (73.6%; 95% CI: 73.5–73.8) and rural (72.9%; 95% CI: 72.7–73.1) areas, and lowest in suburban (70.1%; 95% CI: 70.0–70.5) and urban (69.8%; 95% CI: 69.7–69.9) areas. In contrast, quitting tobacco completely was most probable in urban (9.1%; 95% CI:8.9–9.3) and suburban (9.2%; 95% CI: 9.0–9.4) areas and least probable in town (6.7%; 95% CI: 6.6–6.9) and rural (7.5%; 95% CI: 7.3–7.6) areas. Adjusted predicted probabilities for continued smoking with other added tobacco products were highest in urban (19.8%; 95% CI: 19.7–20.0), followed by suburban (19.1%; 95% CI: 19.0–19.1), rural (18.5%; 95% CI: 18.5–18.5), and town (18.2%; 95% CI: 18.1–18.3) areas. Adjusted predicted probabilities for switching to e-cigarette use were highest in suburban (1.6%; 95% CI:1.5–1.7), followed by town (1.5%; 95% CI: 1.4–1.5), urban (1.3%; 95% CI: 1.3–1.3), and rural (1.1%; 95% CI: 1.1–1.1) areas. Pairwise post-estimation comparisons with Bonferroni p-value corrections indicated no significant differences between any geolocations for the predicted probability of any cigarette use transition (all adjusted p-values >0.05).

**Figure 1:**
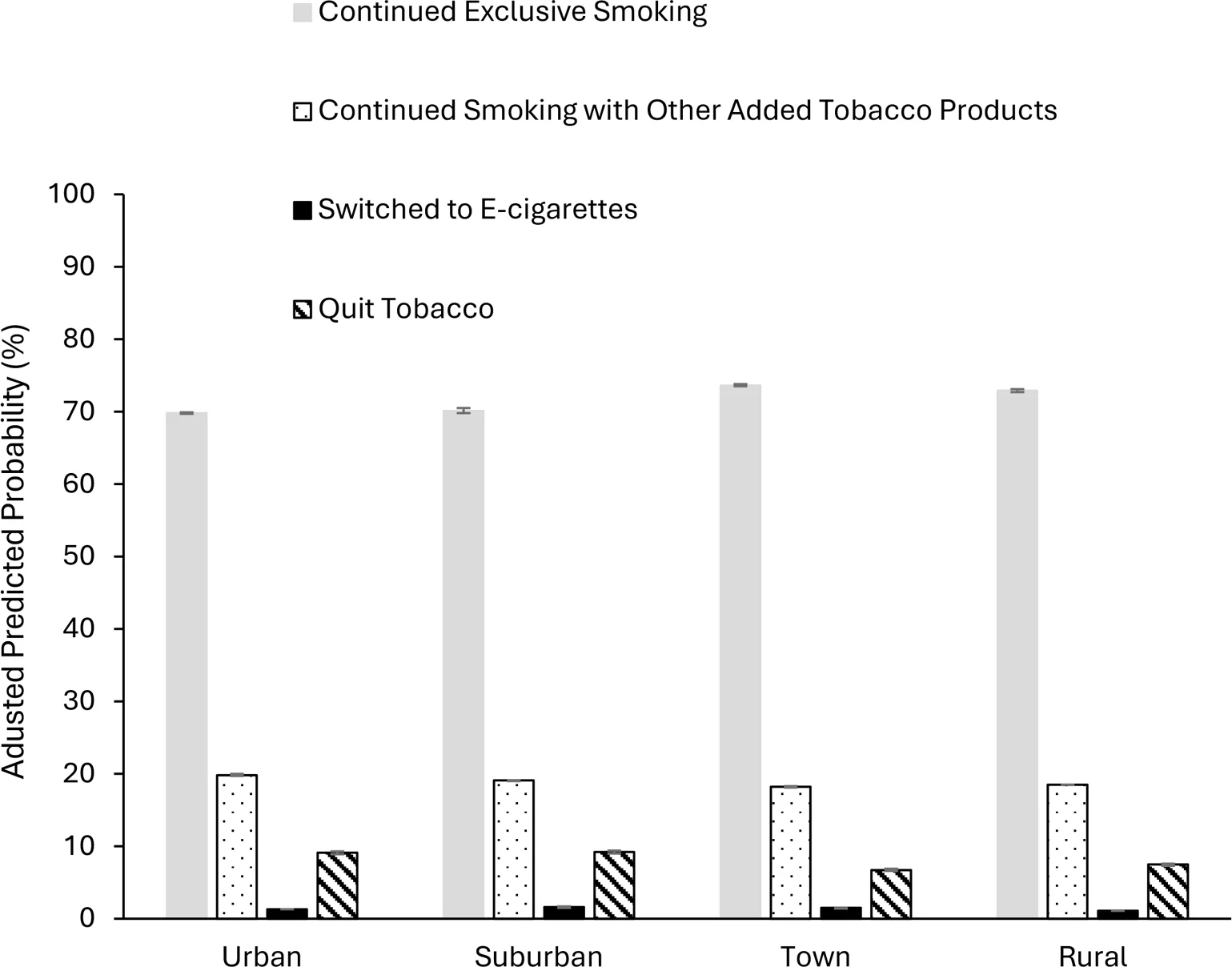
Adjusted predicted probabilities for each cigarette use transition type based on geolocation among adults who exclusively smoked cigarettes in the past 30 days at baseline, Waves 1–6 of the PATH Study, 2013–2021

Predicted probabilities were adjusted for age, sex, race, ethnicity, education level, household income, US region, smoking frequency, cigarette pack-years, minutes to first cigarette upon waking, cigarette risk perceptions, and noticing warnings on cigarette packs in the past 30 days. Pairwise post-estimation comparisons with Bonferroni p-value corrections indicated no significant differences between any geolocations for the predicted probability of any cigarette use transition (all adjusted p-values >0.05). Error bars represent 95% confidence intervals.

### Sensitivity analysis – unweighted sample

In the adjusted unweighted sensitivity analysis (Supplementary file Table 3), compared to urban, only rural (ARRR=1.22; 95% CI: 1.02–1.45; p=0.027) had significantly increased risk of continued exclusive cigarette smoking relative to quitting tobacco.

## Discussion

In this large nationally representative study of US adults, we examined longitudinal associations between geolocation and cigarette use transitions over 1–2 years. We found that overall, most US adults (90.8%) who were smoking cigarettes at a baseline wave continued exclusive cigarette smoking or continued smoking with other added tobacco products 1–2 years later, whereas small fractions switched to exclusive e-cigarette use (1.2%) or quit tobacco completely (8.0%). Compared with adults living in urban areas, adults living in town or rural areas had a higher likelihood of continued exclusive cigarette smoking, even after adjusting for covariates.

Continued exclusive smoking or continued smoking with other added tobacco products accounted for >93% of town and rural wave-pair observations, whereas very few switched to exclusive e-cigarette use (~1%) or quit tobacco completely (~5%). In contrast, urban and suburban cigarette use transitions were more similar to one another, with ~89% continuing to smoke exclusively or with other added tobacco products, ~1% switching to e-cigarette use, and ~9% quitting tobacco completely. In unadjusted analyses, there were significant associations between geolocation and transitions to continued exclusive cigarette smoking (town and rural vs urban), exclusive e-cigarette use (town vs urban), or to continued smoking with other added tobacco products (town and rural vs urban). However, in the adjusted model, associations with exclusive e-cigarette use or continued smoking with other added tobacco products became nonsignificant, suggesting that covariates were responsible for the significant unadjusted associations.

One covariate in particular that was significantly associated with cigarette use transitions and may have confounded the significant unadjusted associations between geolocation and cigarette use transitions was smoking frequency. Daily (vs non-daily) exclusive cigarette smoking at baseline was substantially and significantly associated with all three cigarette use transitions that involved continued tobacco product use compared to quitting tobacco completely in both unadjusted and adjusted models. We also found that, in descriptive analyses, >94% of respondents who exclusively smoked cigarettes daily at baseline continued to smoke either exclusively or with other added tobacco products, whereas 77% of those who smoked non-daily continued to smoke. Additionally, a greater percentage of adults who smoked non-daily reported quitting smoking (21.3%) compared to those who reported daily smoking (4.7%). This finding is consistent with a recent study reporting that adults who smoke non-daily (vs daily) may be more motivated to quit smoking^19^ and highlights an important avenue for future research.

Despite combining six waves of data to increase sample sizes and statistical power, we observed very few transitions from exclusive cigarette smoking to exclusive e-cigarette use and from exclusive cigarette smoking to quitting tobacco completely. Findings align with work suggesting that many adults who smoke cigarettes and use e-cigarettes in an attempt to quit smoking end up using both products^20^. Although using both cigarettes and e-cigarettes may be temporary for some as they transition away from cigarettes toward exclusive e-cigarette use^21^, in our study, such temporary transitions were captured as transitions from exclusive smoking to continued smoking with other added tobacco products. We subsequently conducted an unweighted sensitivity analysis that included adult respondents with partial data who were lost to follow-up to increase the number of wave-pair observations, particularly for the less common transition types (i.e. to exclusive e-cigarette use or quitting tobacco completely). Findings were similar in the unweighted analysis, suggesting that excluding respondents who did not complete all six survey waves (i.e. survey attrition) did not appreciably affect study outcomes.

Overall, findings are consistent with prior studies suggesting the adults in rural areas of the US are less likely to quit smoking cigarettes compared to their urban counterparts^7,8^. The present study expands upon existing literature by using nationally representative data with five wave-to-wave comparisons and a four-category geolocation variable, demonstrating that town and rural areas are similar in prevalence of quitting tobacco. In line with results from a recently published study examining prevalence of past 30-day tobacco use by geolocation^14^, the prevalence of cigarette use transitions for town and rural respondents were more aligned, whereas the prevalence of transitions for urban and suburban respondents was more aligned. Taken together, these findings suggest that rural and small-town residence plays an important role in continued tobacco use. Even though smoking rates have declined dramatically across the US^22^, these data suggest that there are differences in cigarette smoking rates based on rural/town areas. In terms of measurements, these findings also suggest that when smaller cell sizes are problematic, using a cut-point between suburban and town such that town and rural are combined separately from urban and suburban, may be appropriate.

In this study, geolocation was independently associated with transitions to quitting, but not with other transitions (e.g. transitions to exclusive e-cigarette use or adding tobacco products to continued smoking). We also found that the prevalence of switching from exclusive cigarette smoking to continued smoking with other added tobacco products was greater than quitting tobacco completely or switching to exclusive e-cigarette use. A prior study examining associations between urban/rural geolocation and tobacco use prevalence reported that some types of polytobacco use may be more common in rural areas (e.g. cigarettes and smokeless tobacco use) whereas other types may be more common in urban areas (e.g. hookah and cigarillo use)^23^. Given that our study did not distinguish between product combinations and instead combined respondents into a single category if they transitioned from exclusive smoking to continued smoking with other added tobacco products, it is possible that any geolocation differences in transitions to smoking with other product use were ‘washed out’. Future studies may further elucidate associations between geolocation and transitions from cigarette use to specific polytobacco use combinations when adequate sample sizes are available.

Although differences in transitions from exclusive cigarette smoking by geolocation were modest in magnitude, they may have important clinical and public health implications when considered at the population level. Given the substantial burden of tobacco-related morbidity and mortality among adults who smoke^1-3^, even small differences in cessation likelihood across geographical groups may translate into meaningful differences in health outcomes. Clinicians and tobacco treatment programs may consider geographical context as one factor when identifying patients who may benefit from additional cessation support.

### Strengths and limitations

This study has several strengths, including the use of a 4-category geolocation variable and adequate power to assess multiple cigarette use transitions, including transitions from exclusive cigarette smoking to continued smoking with other added tobacco products or to exclusive e-cigarette use.

Despite several strengths, there are also limitations. First, we found differences between our analytic sample and respondents who were excluded from analyses due to missing data on study measures. Given that the excluded sample included a larger percentage of observations that quit tobacco completely and fewer that continued smoking (exclusively or with other added tobacco products), such differences indicate that we may be under- or over-estimating the impact of geolocation on cigarette use transitions. Second, there are numerous classifications for geographical location with widespread use in tobacco research^11-13^, and our analysis included only US adults, so findings may not generalize to other definitions of rurality or youth or adults in other countries. In addition, W1-W4 surveys were conducted annually, whereas W4-W5 and W5-W6 had two years between data collections. Although our analysis controlled for survey wave as a covariate, it is possible that time-interval inconsistencies between surveys could have impacted results. W6 also included a combination of in-person and telephone surveys, whereas the five prior waves included in-person data collection only. Moreover, there are place-associated factors that put people in rural areas of the US at increased risk for tobacco use (e.g. lower tobacco excise taxes greater social norms around tobacco^24,25^; less access to healthcare and smoking cessation support^26^, many of which were not specifically measured or included in this study. It is possible that the associations found in this study could be more directly accounted for by other unmeasured factors, which is a limitation of observational studies in general. Finally, current exclusive cigarette smoking at baseline was defined as P30D use rather than daily smoking. Because we observed differences in cigarette use transitions between adults who smoked daily (vs non-daily), our findings should be interpreted as reflecting transitions among all adults who exclusively smoked cigarettes, including those who smoked non-daily, and may differ from studies restricted to daily smoking.

## Conclusions

Among US adults who exclusively smoked cigarettes at baseline, most continued cigarette smoking over the subsequent 1–2 years, highlighting the persistence of cigarette smoking over time. Rural and town residences were associated with a lower likelihood of quitting tobacco completely compared with urban residence. Findings reinforce prior work suggesting that geolocation significantly impacts tobacco cessation^6-8^ and has implications for greater risk of tobacco-related disease for people living in town and rural areas of the US. A better understanding of the reasons for continued smoking, particularly in town and rural areas, is important to inform targeted interventions and policies.
