# Supplementary material for: Transitions from cigarette smoking by geolocation among US adults: Results from waves 1-6 of the Population Assessment of Tobacco and Health (PATH) study 2013–2021
Source: Tob Induc Dis. 2026 Oct 28;24:10.18332/tid/226559. doi: 10.18332/tid/226559 (PMC13720716; doi:10.18332/tid/226559)
Supplement: Supplementary file 1 [file TID-24-200-s001.pdf]

Tobacco Induced Diseases

Supplementary file

© 2026 Ozga J.E. et al.

DOI:
10.18332/tid/226559

The content has been provided by the author(s) and has not been reviewed, verified, or endorsed by European Publishing. It may not have undergone peer review. The views, opinions, and recommendations expressed are solely those of the author(s) and do not necessarily reflect the position of European Publishing. European Publishing accepts no responsibility or liability for any consequences arising from the use of, or reliance on, this content.

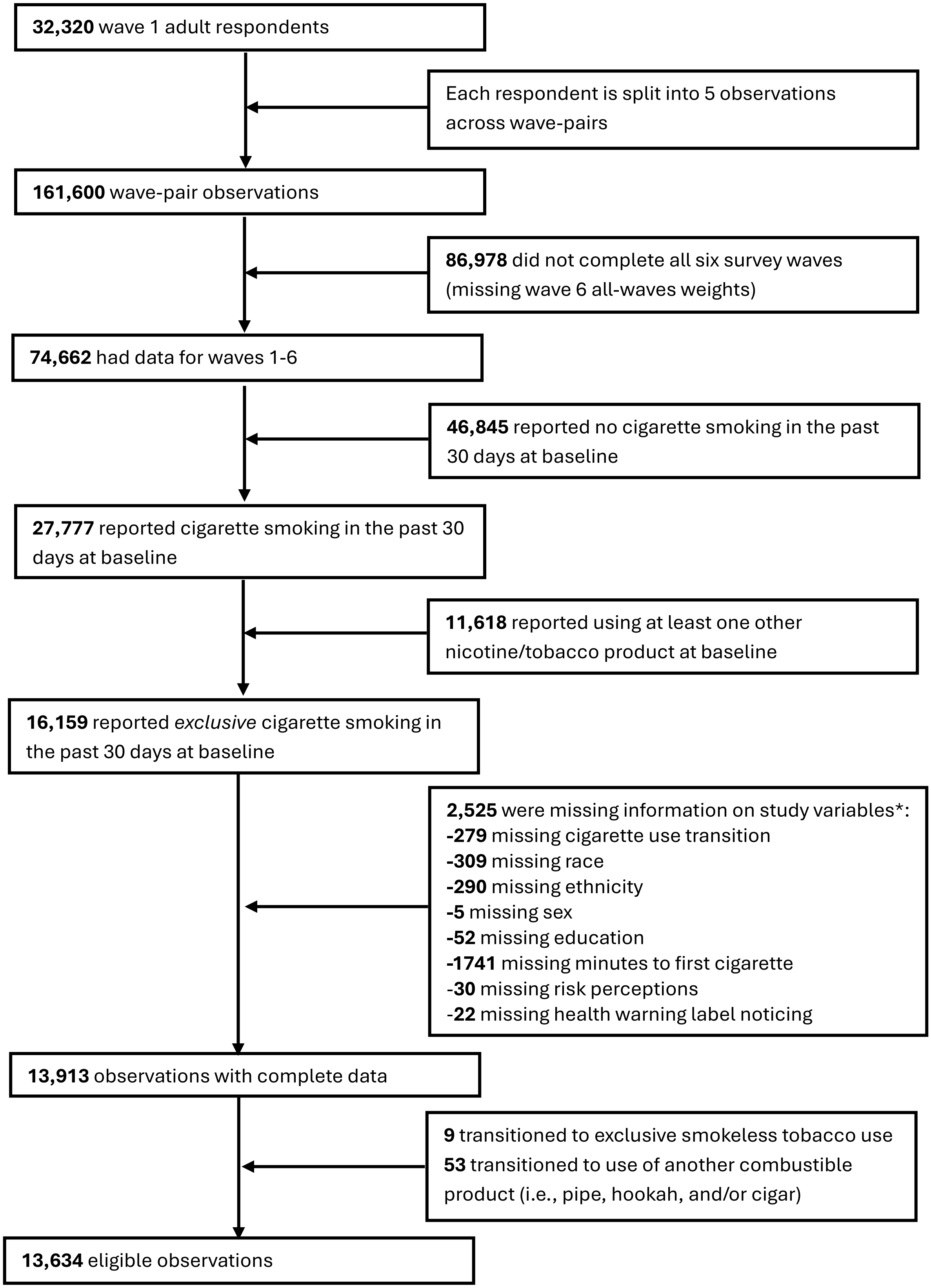

Supplementary file Figure 1. Flow diagram of the weighted analytic sample.

Supplementary file Table 1. Comparison between reduced sample after excluding observations that were missing on one or more study variables and excluded sample only among adults from Waves 1-6 of the PATH Study who exclusively smoked cigarettes at baseline.

	Reduced Sample (Obs=13,634)[1]	Excluded Sample (Obs=2,525)[1]	p value
	Weighted Column % or (mean) (95% CI)[2]		
**Cigarette Use Transition**			<0.001
Continued exclusive cigarette smoking	73.7 (72.8, 74.6)	45.5 (42.1, 49.0)	
Continued smoking with other added tobacco products	17.1 (16.3, 18.0)	12.5 (11.1, 14.2)	
Switch to exclusive e-cigarette use	1.2 (1.0, 1.4)	2.1 (1.5, 2.9)	
Quit tobacco completely	8.0 (7.3, 8.7)	39.8 (36.5, 43.2)	
**Rurality**			<0.001
Urban	28.3 (24.7, 32.2)	34.9 (30.4, 39.6)	
Suburban	32.3 (27.9, 37.0)	36.7 (32.2, 41.5)	
Town	12.1 (9.2, 15.7)	10.1 (7.7, 13.1)	
Rural	27.3 (23.1, 32.0)	18.4 (15.7, 21.4)	
**U.S. Region**			<0.001
Northeast	18.4 (16.5, 20.4)	17.1 (14.6, 20.0)	
Midwest	23.6 (22.0, 25.4)	19.2 (17.1, 21.5)	
South	40.2 (37.6, 42.9)	38.0 (34.9, 41.3)	
West	17.8 (15.9, 19.8)	25.7 (22.6, 29.0)	
**Age**			<0.001
18-24	6.1 (5.5, 6.7)	13.5 (12.0, 15.2)	
25-39	32.6 (31.0, 34.2)	32.0 (29.1, 35.1)	
40-54	31.2 (29.5, 33.1)	27.5 (24.7, 30.5)	
55+	30.1 (28.3, 31.9)	26.9 (24.4, 29.6)	
**Sex**			0.156
Male	49.1 (47.5, 50.6)	51.3 (48.4, 54.1)	
Female	51.0 (49.4, 52.5)	48.8 (45.9, 51.7)	
**Race**			<0.001
White	78.9 (77.4, 80.3)	70.1 (66.8, 73.1)	
Black	14.7 (13.6, 16.0)	19.7 (17.4, 22.1)	
Other/Multiple	6.3 (5.6, 7.2)	10.3 (8.0, 13.1)	
**Ethnicity**			<0.001
Hispanic	11.3 (10.3, 12.5)	28.4 (25.4, 31.5)	
Not Hispanic	88.7 (87.6, 89.7)	71.6 (68.5, 74.6)	
**Education**			<0.001
<High school	26.5 (25.1, 28.0)	26.9 (24.3, 29.7)	
High school degree or GED	27.8 (26.3, 29.4)	25.7 (23.0, 28.7)	
Some college or Associate's degree	33.1 (31.5, 34.7)	27.0 (24.4, 29.7)	
Bachelor's or advanced degree	12.6 (11.4, 13.8)	20.4 (17.9, 23.2)	
**Household income**			<0.001
$100,000+	7.3 (6.3, 8.5)	10.3 (8.7, 12.2)	
$50,000-$99,999	20.0 (18.7, 21.3)	17.9 (15.9, 20.1)	

$25,000-$49,999	24.5 (23.4, 25.6)	19.8 (17.7, 22.2)	
<$25,000	42.6 (40.8, 44.3)	44.3 (41.5, 47.2)	
**Smoking Frequency**			<0.001
Daily	80.2 (78.9, 81.5)	21.0 (18.4, 23.9)	
Non-daily	19.8 (18.5, 21.1)	79.0 (76.2, 81.6)	
**Mean Cigarette Pack-Years**	(21.2) (20.3, 22.0)	(56.8) (54.1, 59.5)	<0.001
**Mean Minutes to First Cigarette upon Waking**	(95.3) (89.2, 101.5)	(137.0) (110.3, 163.7)	0.002
**Cigarette Risk Perception**			<0.001
Not at all, Slightly, or Somewhat Harmful	27.5 (26.3, 28.6)	17.3 (15.4, 19.4)	
Very or Extremely Harmful	72.6 (71.4, 73.7)	82.7 (80.6, 84.6)	
**Noticed warnings on cigarette packs in the past 30 days**	77.0 (75.7, 78.2)	66.0 (63.0, 68.9)	<0.001

[1] The reduced sample includes all adults who completed Waves 1-6 surveys, exclusively smoked cigarettes during at least one baseline wave, and were not missing data on any study measures. The reduced sample is the analytic sample. The excluded sample includes all adults from Waves 1-6 who exclusively smoked cigarettes during at least one baseline and who were missing data on one or more study measures.

[2] Weighted using the PATH Study Wave 6 all-waves survey weights.

CI=confidence interval; Obs=number of observations.

P values come from chi-square tests for categorical variables and one-way analysis of variance (ANOVA) for continuous variables.

Supplementary file Table 2. Adjusted[1] predicted probabilities for each tobacco use transition type based on geolocation among adults in Waves 1-6 (2013-2021) of the Population Assessment of Tobacco and Health (PATH) Study who exclusively smoked cigarettes in the past 30 days at baseline.

	**Transition Type from Baseline to Follow-up**			
	**Continued Exclusive Smoking (Obs=10,080)**	**Continued Smoking with Other Added Tobacco Products (Obs=2,392)**	**Switched to E-cigarettes (Obs=166)**	**Quit Tobacco Completely (Obs=996)**
	**% (95% confidence interval)**			
Urban	69.8% (69.7, 69.9)	19.8% (19.7, 20.0)	1.3% (1.3, 1.3)	9.1% (8.9, 9.3)
Suburban	70.1% (69.8, 70.5)	19.1% (19.0, 19.1)	1.6% (1.5, 1.7)	9.2% (9.0, 9.4)
Town	73.6% (73.5, 73.8)	18.2% (18.1, 18.3)	1.5% (1.4, 1.5)	6.7% (6.6, 6.9)
Rural	72.9% (72.7, 73.1)	18.5% (18.5, 18.5)	1.1% (1.1, 1.1)	7.5% (7.3, 7.6)

[1]Adjusted for age, sex, race, ethnicity, education, household income, U.S. region, smoking frequency, cigarette pack-years, minutes to first cigarette upon waking, cigarette risk perceptions, and noticing warnings on cigarette packs in the past 30 days.

Note: Pair-wise post-estimation comparisons with Bonferroni p value corrections indicated no significant differences between any geolocations for the predicted probability of any cigarette use transition (all adjusted p’s > 0.05).

Supplementary file Table 3. Adjusted[1] unweighted multinomial logistic regressions examining associations between geolocation and cigarette use transitions (reference = quitting all tobacco; Obs=1,536) among adults in Waves 1-6 (2013-2021) of the Population Assessment of Tobacco and Health (PATH) Study who exclusively smoked cigarettes in the past 30 days at baseline.

	**Continued Exclusive Smoking (Obs=14,972)**	**Continued Smoking with Other Added Tobacco Products (Obs=3,847)**	**Switch to E-cigarette Use (Obs=273)**
	aRRR (95% CI)		
Urban	Ref	Ref	Ref
Suburban	0.93 (0.81, 1.08)	0.95 (0.81, 1.12)	1.17 (0.84, 1.64)
Town	1.07 (0.87, 1.32)	0.92 (0.72, 1.16)	1.01 (0.64, 1.61)
Rural	**1.22 (1.02, 1.45)**	1.07 (0.88, 1.30)	1.09 (0.74, 1.62)

Obs=number of observations. aRRR=adjusted relative risk ratio. CI=confidence interval. Bolded values denote statistical significance, $p<0.05$.

[1]Adjusted for U.S. region, age, sex, race, ethnicity, education, household income, smoking frequency, cigarette pack-years, minutes to first cigarette upon waking, cigarette risk perceptions, and noticing warnings on cigarette packs in the past 30 days at baseline.
